# Exploitation of Autophagy Inducers in the Management of Dementia: A Systematic Review

**DOI:** 10.3390/ijms25021264

**Published:** 2024-01-19

**Authors:** Maria Tiziana Corasaniti, Giacinto Bagetta, Pierluigi Nicotera, Sabatino Maione, Paolo Tonin, Francesca Guida, Damiana Scuteri

**Affiliations:** 1 Department of Health Sciences, University “Magna Graecia” of Catanzaro, 88100 Catanzaro, Italy; mtcorasa@unicz.it; 2 Pharmacotechnology Documentation and Transfer Unit, Preclinical and Translational Pharmacology, Department of Pharmacy, Health and Nutritional Sciences, University of Calabria, 87036 Rende, Italy; giacinto.bagetta@unical.it; 3 German Center for Neurodegenerative Diseases (DZNE), 53127 Bonn, Germany; pierluigi.nicotera@dzne.de; 4 Division of Pharmacology, Department of Experimental Medicine, University of Campania “L. Vanvitelli”, 80138 Naples, Italy; sabatino.maione@unicampania.it (S.M.); franc.guida@gmail.com (F.G.); 5 Laboratory of Biomolecules, Venoms and Theranostic Application, Institute Pasteur de Tunis, Université Tunis El Manar, Tunis 1002, Tunisia; 6 Regional Center for Serious Brain Injuries, S. Anna Institute, 88900 Crotone, Italy; patonin18@gmail.com

**Keywords:** dementia, autophagy, autophagy inducers, metformin, resveratrol, masitinib, TPI-287

## Abstract

The social burden of dementia is remarkable since it affects some 57.4 million people all over the world. Impairment of autophagy in age-related diseases, such as dementia, deserves deep investigation for the detection of novel disease-modifying approaches. Several drugs belonging to different classes were suggested to be effective in managing Alzheimer’s disease (AD) by means of autophagy induction. Useful autophagy inducers in AD should be endowed with a direct, measurable effect on autophagy, have a safe tolerability profile, and have the capability to cross the blood–brain barrier, at least with poor penetration. According to the PRISMA 2020 recommendations, we propose here a systematic review to appraise the measurable effectiveness of autophagy inducers in the improvement of cognitive decline and neuropsychiatric symptoms in clinical trials and retrospective studies. The systematic search retrieved 3067 records, 10 of which met the eligibility criteria. The outcomes most influenced by the treatment were cognition and executive functioning, pointing at a role for metformin, resveratrol, masitinib and TPI-287, with an overall tolerable safety profile. Differences in sample power, intervention, patients enrolled, assessment, and measure of outcomes prevents generalization of results. Moreover, the domain of behavioral symptoms was found to be less investigated, thus prompting new prospective studies with homogeneous design. PROSPERO registration: CRD42023393456.

## 1. Introduction

Some 57.4 million people all over the world are affected by dementia, and this number is expected to triple to 152.8 by 2050, with a female-to-male ratio of 1.67 [1]. Research effort in the finding of disease-modifying drugs is generating a wide number of clinical studies. Autophagy is a process evolutionarily conserved from yeast to humans [2], is subjected to alterations during aging, and is thus related to aging-related pathological conditions such as neurodegeneration and dementia [3,4]. The role of the induction of autophagy in the pathophysiology of dementia deserves deep investigation. Several drugs able to induce autophagy have been shown to reduce signs of neurodegeneration, acting at the loss of proteostasis due to the accumulation of protein aggregates [5,6]. In support of the role of autophagy in neurodegeneration, the latter diseases were found to be associated with increased autophagic vacuoles; this occurs in Alzheimer’s disease (AD) [7], in which autophagosome-like bodies were found to accumulate preventing autolysosomes maturation both in preclinical [8] and in clinical models [9]. Therefore, impairment in the pathway of basal macroautophagy—which needs to be tightly regulated, since it is involved in cell proliferation and death [10]—can underlie the aberrant synthesis of toxic amyloid beta (Aβ) [11] (assembling in monomers, oligomers and fibrils [12]), making this process of self-digestion a target for the treatment of dementia and of disorders associated to AD [13]. Incidentally, early endosomes were detected in up to 32-times larger volumes than normal size and morphometry in human pyramidal neurons of sporadic AD subjects at early stages, but not in neurons of patients suffering from other neurodegenerative diseases [14]. Therefore, dysfunction in multiple steps of the autophagic process is supposed to represent one of the pathways involved in the development of AD [15,16]. Another pathological hallmark of AD consists of filamentous inclusions in pyramidal neurons, known as neurofibrillary tangles (NFTs), made of hyperphosphorylated microtubule-associated protein tau [17]. There is evidence suggesting that not fully functional, enlarged, and delayed in turn-over autophagosomes containing Aβ can lead to its extracellular accumulation due to impaired clearance [18]. Also, enlarged autolysosomes induced by treatment of an AD mouse model with the anaesthetic sevoflurane were proposed as a marker for prediction of neurotoxicity in this disease [19]. Therefore, dysfunction of autophagosomes and reduced autophagic flux are supposed to trigger AD, and autophagy inducers might be therapeutic for the treatment of this disease. Using the approach of drug repurposing, or repositioning, several drugs belonging to different classes, also identifiable through machine learning [20], were suggested to be effective in managing AD by means of action on autophagy; e.g., anti-viral and anti-cancer drugs [21,22,23]. Drugs supposed to be useful autophagy inducers in AD should produce a direct, measurable effect on autophagy with tolerable toxicity, and prove to be able to cross the blood–brain barrier (BBB), at least with poor penetration. Repurposed drugs meeting these requirements include some inhibitors of the mammalian target of rapamycin (mTOR; e.g., rapamycin and temsirolimus) as anti-cancer drugs, the activators of the negative mTOR regulator AMP-activated protein kinase (AMPK; e.g., the anti-diabetic metformin), the anti-epileptic carbamazepine used in the treatment of trigeminal neuralgia, and some anti-cancer drugs. Indeed, apart from the reported mTOR dysregulation in models of cognitive decline [24], evidence in favor of the role of mTOR as an inhibitor of autophagy, and in the decrease of the clearance of Aβ and of NFTs was provided by the use of rapamycin, vinblastine, metformin, temsirolimus, carbamazepine, and dactosilib in transgenic AD murine models [25,26,27,28,29,30,31,32,33]. This also occurred for resveratrol [34,35], and in cell lines for small molecules specifically synthesized as AMPK activators to enhance autophagy, identified through a library screening for similarity with resveratrol [36]. Furthermore, the tyrosine kinase inhibitor masitinib was investigated as add-on therapy to standard care for the treatment of mild to moderate AD in the clinical trial NCT00976118 (ClinicalTrials.gov), proving effective on cognitive decline with acceptable tolerability [37]. TPI 287 is an abeotaxane that could be useful to induce autophagy in AD since it crosses BBB efficiently [38]. Finally, anti-hyperlipidemic drugs such as gemfibrozil or statins were suggested to act on AD through autophagy induction in AD models [39,40]. Patients suffering from dementia develop symptoms other than cognitive impairment: the latter include a heterogeneous set of psychological reactions, psychiatric symptoms, and behavioral disorders common to all types of dementia and very frequent in AD, known as behavioral and psychological symptoms of dementia (BPSD), with an incidence of 99% of the population affected by AD [41]. A biopsychosocial model was proposed that attributes BPSD to the interaction among the individual’s biology, previous experiences, and the current environment, also correlated with alterations in cholinergic, noradrenergic, dopaminergic, serotoninergic, and glutamatergic neurotransmissions [42]. A previous systematic review investigated the role of mitophagy inducers/modulators in AD, pointing at the lack of a true target for the effects reported for these drugs [43]. Therefore, the purpose of the present systematic review was to appraise the measurable effectiveness of autophagy inducers in the improvement of cognitive decline and neuropsychiatric symptoms due to dementia in clinical trials (prospective studies) and real-world evidence (retrospective studies). The results were reported according to the Preferred Reporting Items for Systematic reviews and Meta-Analyses (PRISMA) 2020 recommendations [44]. This systematic review, for which meta-analysis was planned in case of the retrieval of homogeneous and comparable data, was registered in the National Institute for Health Research (NIHR) International prospective register of systematic reviews (PROSPERO) with the number CRD42023393456.

## 2. Materials and Methods

### 2.1. Objectives, Protocol, and Registration

The process of database screening, selection of results, and extraction of data was performed in agreement with PRISMA 2020 recommendations [44,45]. Based on PRISMA, the research question is formulated as a question based on participants/population, interventions, comparisons, outcomes, and study design, known as PICOS. The participants are patients affected by dementia of any etiology. The intervention is represented by autophagy inducers administered in any dose and route. Studies deemed to be eligible must be clinical trials or retrospective and real-world evidence aimed at evaluating the effectiveness of the intervention over a comparator consisting of placebo/no treatment or an active control used in the treatment of dementia. Drugs used need to prove to be able to cross the BBB for the study to be considered eligible. The primary outcome consists of improvement in cognitive decline and neuropsychiatric symptoms due to dementia. Preclinical studies, reviews, book chapters, and congress proceedings are not eligible study designs. No restriction about time of publication was set, and the search was conducted from database inception. Studies in which the drug tested was not reported to be able to cross the BBB and studies not available as full text in English were excluded. The protocol was set before the beginning of literature search and registered in PROSPERO (CRD42023393456). 

### 2.2. Information Sources

PubMed/MEDLINE, Scopus, and WOS were inspected for studies published from database inception to the date of last search on 24 October 2023. The search was performed by two independent members of the review committee for records matching the strategy strings. The process of removal of duplicate records was carried out using a reference manager software (EndNote X7, Clarivate, London, UK).

### 2.3. Search Strategy

The following medical and subject heading (MeSH) terms were used in combination within search strings for search on PubMed/MEDLINE: (“Dementia”[Mesh]) AND “Autophagy”[Mesh]; the keywords used in combination are “dementia”, “autophagy inducers”, “drugs”, “autophagy”, “rapamycin”, “temsirolimus”, “metformin”, “dactosilib”, “resveratrol”, “masitinib”, “abeotaxane”, “gemfibrozil”, “statins”, “carbamazepine”. Scopus was searched for. Article Title, Abstract, Keywords, and WOS for all fields. The search was conducted with a high sensitivity/recall search strategy [46]. Two requestors independently searched the databases, while a third member of the review team (reviewer) checked the accuracy of the spelling of search strings and to answer to the PICOS question, according to the evidence-based guideline for Peer Review of Electronic Search Strategies (PRESS) for systematic reviews (SRs) [46,47].

### 2.4. Study Selection

Two independent members of the review committee assessed the eligibility of the retrieved results to minimize the risk of missing relevant records. The extraction of data was based on searched terms in the text, tables, or graphs. Data extracted include: the report (author and year); the study design and sample size; the participants, based on type of dementia; the research design with sampling, treatment assignment, any randomization, allocation and concealment mechanisms; the intervention type, timing, dose, and study duration; and the results in terms of primary and secondary outcome measures of the systematic review. The title and abstract, and subsequently the full text, were screened. The reference lists of relevant papers was inspected for additional studies potentially missed in the database search. Any disagreement was planned to be solved by consensus or by consultation with a third member of the team.

### 2.5. Data Synthesis, Assessment of the Risk of Bias, and Critical Appraisal

The synthesis of the results was conducted according to the Cochrane Consumers and Communication Review Group guidelines [48]. The risk of bias (RoB) within and among the studies and the assessment of the certainty of evidence [49], in agreement with the PRISMA 2020 statement [45], was planned to be conducted independently by two members of the review committee. The tools selected are the revised Cochrane risk of bias tool RoB2 for randomized clinical trials [50] and the ROBINS-I tool for studies not randomized [51]. However, the study that resulted retrospective after the analysis, had to be excluded from the assessment of the risk of bias. The graphical summary of the assessment of the RoB as a traffic-light plot and weighted bar plots was made using the Cochrane robvis visualization tool [52]. 

### 2.6. Statistical Analysis and Effect Measures

Standardized mean differences (SMD), inverse variance and/or risk ratio (RR), and 95% confidence intervals (CI)—based on the continuous or dichotomous nature of the variables resulting from primary and secondary outcome measures—were planned to be calculated through the Cochrane Review Manager 5.4.1 (RevMan5.4.1; Copenhagen: The Nordic Cochrane Center, The Cochrane Collaboration). The heterogeneity of the results subjected to meta-analysis was planned to be evaluated using the random effect model [53] and the Higgins I^2^ value [54]. Publication bias was planned to be assessed by means of funnel plot asymmetry [55]. However, the differences in the measures of outcomes and in the selection of the study population did not permit a meta-analysis to be performed.

## 3. Results

### 3.1. Selection of the Studies

**Figure 1:**
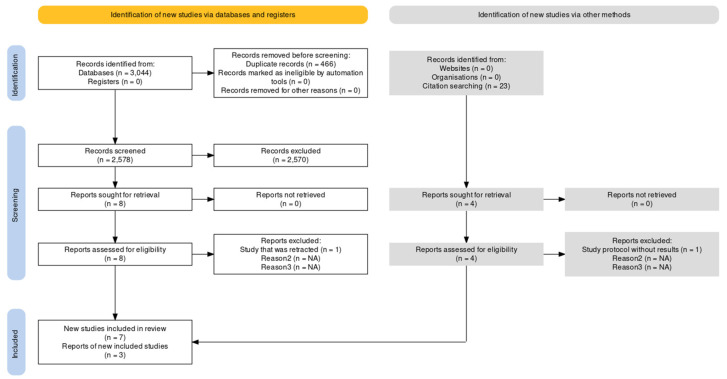
Process of selection of records in agreement with the Preferred Reporting Items for Systematic Reviews and Meta-Analyses (PRISMA) 2020 flow diagram, created using the web-based Shiny app [86].

The search was conducted screening PubMed/MEDLINE, Scopus and WOS since their ince ption up to 24 October 2023, the date of the last search. The search retrieved 3044 total records from the following databases: 1301 records were retrieved from PubMed/MEDLINE, 1606 from Scopus, and 137 from WOS. Additional studies retrieved from screening of the list of references amount to 23. Therefore, the sum of records found from database searching and from reference list screening is 3067. After the removal of duplicates, 2601 records (2578 from databases + 23 from lists of references) were left to screen for eligibility. Only studies investigating drugs that present an effect of autophagy induction and the capability to cross the BBB maintaining a concentration compatible with clinical efficacy were included. Since low nanomolar native resveratrol was detected in cerebrospinal fluid (CSF), penetration of the central nervous system (CNS) is supported [56], thus clinical trials investigating the effects of resveratrol were deemed eligible for inclusion in the analysis. TPI-287 (abeotaxane) is a BBB-penetrable microtubule stabilizer; therefore, it was a treatment included in this study [57]. The natural polyamine spermidine was included since its accumulation in the forebrain parenchyma after ischemia was reported [58], suggesting its increased capability to cross the BBB in neurological and pathological states [59] in spite of quite limited physiological transport [60,61]. Studies investigating the effect of masitinib were included because masitinib was reported to extensively penetrate the BBB and to modulate BBB and blood-spinal cord barrier permeability [62]. Grape seed proanthocyanidins were suggested to induce autophagy [63], and although they can affect the cytokines penetration of BBB [64], their uptake is not reported, thus causing the exclusion of the study investigating the efficacy of grape consumption [65]. Metformin was included, since it is reported to cross BBB [66], as well as pioglitazone [67]; nevertheless, the antidiabetic dose of the latter peroxisome proliferator-activated receptor-γ (PPAR-γ) agonist does not obtain a therapeutic concentration in the brain [68]; thus, studies using pioglitazone were excluded. On the contrary, studies focusing on rosiglitazone were excluded since it does not readily cross BBB in rodents [69,70], its low brain uptake highlighted [71,72]. Finally, liraglutide was included, as it is proven to cross the BBB, to be useful in a mice model of AD [73], and to enhance autophagy [74,75]. One study involved the use of the mixed antioxidant supplement Twendee X [76], but its capability to reach the brain parenchyma is still debated [77], thus leading to exclusion of this result as it occurs for studies assessing the efficacy of selenium and nicotinamide supplementation [78,79,80,81,82]. The CNS penetration and uptake of a therapeutic dose of all these drugs, through the treatment regimen used, is an aspect of the utmost importance that deserves further investigation. After the screening of the title and abstract; book chapters, proceedings, reviews, in vivo and in vitro studies, as well as out of scope studies, case reports and post-mortem brain studies were excluded, leaving 12 (8 from databases + 4 from lists of references) records to screen in full. Full text screening left 10 results to be included in the analysis. In fact, the study by Fang et al., 2022 [83] had to be excluded although it met inclusion criteria, because significant concerns were identified with the peer review process that led to the retraction of the article [84], and the study by Egefjord et al., 2012 [85] was a protocol without results. The selection of the eligible studies is illustrated in Figure 1.

### 3.2. Synthesis of the Extracted Data and Critical Appraisal

#### 3.2.1. Metformin

The efficacy of metformin on the endpoints object of the analysis was examined in the studies by Koenig et al., 2017 [87] and Luchsinger et al., 2016 [88]. 

The study by Koenig et al., 2017 (NCT01965756) [87] is a deep-phenotyping, randomized, double-blinded, placebo-controlled crossover pilot study investigating the effect of an eight-week long treatment with metformin on cognitive, executive, learning, and memory tests, cerebrospinal fluid (CSF) biomarkers, and on magnetic resonance imaging (MRI) parameters of *n* = 20 non-diabetic subjects suffering from mild cognitive impairment or mild AD. The trial was approved by the Human Subjects Institutional Review Board at the University of Pennsylvania. The University of Pennsylvania Health System (UPHS) research pharmacy was responsible for keeping drug preparation blinded, and providing an opaque placebo capsule, identical in appearance to the capsule of intervention. Also, investigators were blind until trial completion. Randomization to each group was conducted using a simple computer-generated randomization table. Therefore, no bias arises in these domains. The patients included in the trial had to fulfill the characteristics as follows: aged 55–80 years, without history of diabetes or pre-diabetes, with diagnosed mild cognitive impairment (MCI) or early AD [graded as Cognitive Dementia Rating (CDR)-Global score ≤ 1.0 and Mini-Mental State Examination (MMSE) score > 19]; presenting at least one positive biomarker of AD, merging from CSF analysis, positron emission tomography (PET), or amyloid scan; baseline Geriatric Depression Scale (GDS) total < 6; modified Hachinski Ischemic Score < 4; any acetylcholinesterase inhibitors on a stable dose for at least 2 months before baseline; without any other CNS, pancreatic, liver, or renal or history of substance abuse/dependence within the past two years; contraindication to participation in MRI or lumbar puncture; and not treated with any other anticholinergic medications. The cognitive assessment was repeated at weeks 0, 8, and 16, consisting of the following tests: cognitive subscale, immediate learning, and delayed recall of the word list of the Alzheimer’s Disease Assessment Scale (ADAS-Cog). The computerized Cambridge Neuropsychological Test Automated Battery (CANTAB) was performed to assess learning, executive functioning (Trails-B on backwards Digit Span), attention, language, and motor speed. The assessment of depression was performed through the Geriatric Depression Scale (GDS). The Dementia Severity Rating Scale (DSRS) was used for the evaluation of functioning. Therefore, no bias in the measurement of the outcome is detectable. Baseline characteristics do not show statistically significant differences between the two groups (group 1: placebo followed by metformin; group 2: metformin followed by placebo), not pointing at bias. A 15% drop-out rate with a two-sided paired *t*-test with alpha = 0.05 and power = 0.80 was calculated. The results demonstrated that the only statistically significant treatment effect was reported for executive functioning (Trails-B: *p* < 0.05). Metformin was well-tolerated and its steady-state concentration levels in CSF amounted to 95.6 ng/mL. No statistically significant treatment effect for functional neuroimaging was observed. No serious adverse events were reported during the trial to be associated with the use of metformin. No bias of selective reporting occurred. 

The study by Luchsinger et al., 2016 (NCT00620191) [88] was a double-blind, placebo-controlled, randomized, pilot trial enrolling *n* = 80, 55–90 year-old patients suffering from amnestic mild cognitive impairment (aMCI), in which memory is impaired [89] according to Petersen criteria [90], without treated diabetes and who were not overweight or obese (body mass index, BMI ≥ 25), randomized 1:1 to receive identical-appearing pills of metformin (maximum dose 1000 mg twice a day) or a matching placebo. Therefore, no bias arises in this domain. Metformin was titrated up to the highest tolerated dose without side effects weekly from 500 mg once a day to 1000 mg twice a day in a 4-week period. The allocation concealment was obtained through randomly alternating block sizes. The change of inclusion criteria at the beginning of the study can raise some concerns in terms of bias. The primary outcomes included changes from baseline to month 12 in total recall of the Bushcke Selective Reminding Test, and of ADAS-Cog. Other measures included: the Alzheimer’s Disease Cooperative Study (ADCS); Clinical Global Impression of Change for Mild Cognitive Impairment (CGIC-MCI); the logical memory II delayed paragraph recall sub-test of the Wechsler Memory Scale Revised (WMS-R); the MMSE; the Neuropsychiatric Inventory Questionnaire (NPI-Q) [49]; and the digit span backwards. The primary imaging outcome consisted of changes from the baseline to month 12 of relative glucose uptake (rCMRgl) in the posterior cingulate-precuneous measured by non-quantitative brain ^[18]^F-labeled 2-deoxy-2-fluoro-D-glucose (FDG) positron emission tomography (PET) with magnetic resonance imaging (MRI) co-registration. Therefore, no bias in outcome measures occurred. Also, plasma A β42 levels were measured, and genotyping of APOE polymorphisms rs7412 and rs429358 was performed. Some confounding biases arise from the evidence that the only statistically significant baseline difference between the groups was the ADAS-Cog score, which is better for the metformin group. No serious adverse events related to metformin were reported. In the study, 7.5% of patients dropped out for gastrointestinal symptoms. No bias of selective reporting occurred. There were no statistically significant differences between metformin and placebo for the memory, cognitive, and neuropsychiatric outcome measures, or for the other outcomes. After adjusting for baseline ADAS-Cog, changes in the total recall of the Selective Reminding Test favored the metformin group (9.7 ± 8.5 vs. 5.3 ± 8.5; *p* = 0.02). 

#### 3.2.2. Antitumoral Drugs

The study by Piette et al., 2011 [37] (NCT00976118), as well as the study by Dubois et al., 2023 [91] (AB09004) investigated the effect of masitinib. The study by Tsai et al., 2019 (NCT019666666 and NCT02133846) [57] evaluated the effect of the microtubule stabilizer TPI-287.

The 24-week multicenter (12 study centers across France), double-blind, randomized, placebo-controlled, parallel-group, phase II study by Piette et al., 2011 [37] investigated the efficacy of masitinib as an adjunct to a cholinesterase inhibitor and/or memantine, thus using an appropriate study design, and recruiting patients affected by mild-to-moderate AD. The diagnosis was based on the Diagnostic and Statistical Manual of Mental Disorders IV criteria and the National Institute of Neurological and Communicative Disorders and Stroke-Alzheimer’s Disease and Related Disorders Association criteria, and graded according to baseline MMSE score between 12 and 26 and baseline Clinical Dementia Rating (CDR) of 1 or 2. Randomization consisted of assigning patients to receive masitinib (*n* = 26) with a starting dose of 3 or 6 mg/kg *die* or placebo (*n* = 8), administered twice daily for 24 weeks. Moreover, the treatment with cholinesterase inhibitors (donepezil, rivastigmine, or galantamine) and/or memantine had to be stable for at least 6 months or 3 months, respectively. Patients deemed to be excluded were those presenting the following conditions: delusions, delirium, uncontrolled depression, evidence of psychosis and/or use of antipsychotic drugs, a history of significant psychotic/psychiatric disorders, active infection, treatment with a cognitive enhancer, treatment with an investigational drug for the last 4 weeks of inclusion, or had a history of poor compliance. The primary endpoint was the ADAS-Cog mean difference at week 24 from the baseline defined by a blind data review committee prior to unblinding. Secondary outcomes included the following assessments: the Alzheimer’s Disease Cooperative Study Activities of Daily Living Inventory (ADCS-ADL); the Clinician’s Interview-Based Impression of Change-plus caregiver input (CIBIC-Plus); the MMSE and the CDR; and safety assessment through physical examinations, vital signs, clinical laboratory evaluations, and monitoring of adverse events. Therefore, no bias in outcome measures occurred. There were no baseline significant differences, thus suggesting an absence of selection bias. The results demonstrate that the decline of cognitive functions according to ADAS-Cog was significantly higher in the placebo group in comparison with the masitinib group after 12 and 24 weeks (50% vs. 6% for both; *p* = 0.040 and *p* = 0.046, respectively). Furthermore, a statistically significant difference in ADASCog in comparison with baseline was shown between the masitinib and placebo groups at week 12 (*p* = 0.016) and at week 24 (*p* = 0.030). Incidentally, better cognitive improvement according to a decrease in ADAS-Cog score ≥ 4 was observed in the higher masitinib dose (6 mg/kg/day), supporting 6 mg/kg/day as the best starting dose. The mean absolute change values of MMSE were as follows: at week 12, masitinib (0.1 ± 2.5, *n* = 17) and placebo (−2.1 ± 2.5, *n* = 7), *p* = 0.047; at week 24, masitinib (−0.1 ± 4.3, *n* = 16) and placebo (−3.3 ± 3.3, *n* = 7), *p* = 0.031. Also, the CIBIC-Plus score became worse in a lower proportion of patients in the masitinib group in comparison with the placebo group, and 15/16 (94%) masitinib patients remained stable or improved relative to the CDR baseline compared with 5/7 placebo patients (71%) after 24 weeks. Adverse effects were more commonly reported in the masitinib group rather than the placebo group, but they were mostly of mild-to-moderate intensity and transitory. One patient from the masitinib group discontinued donepezil on the first day of the study and was withdrawn on day 29 due to this major protocol deviation. No bias of selective reporting occurred. 

The following study AB09004 by Dubois et al., 2023 [91] was a randomized, double-blind, two parallel-group (four-arm), placebo-controlled trial conducted according to the Declaration of Helsinki, Good Clinical Practice (GCP) guidelines, and national regulations. The trial protocol was approved by the appropriate Independent Ethics Committee/Institutional Review Board of all participating sites (including the ethics committee of General Hospital of Thessaloniki ‘George Papanikolaou’), and all subjects provided informed consent. An independent data monitoring committee periodically reviewed blinded patient safety and efficacy data. Therefore, all these procedures prevented bias sources in these domains. Patients over 50 years, diagnosed with mild-to-moderate, probable AD, and with MMSE = 12–25 were randomized (1:1) to receive oral masitinib 4.5 mg/kg/day, as two intakes, or placebo in the first group. In the second, independent, parallel group, patients were randomized (2:1) to masitinib at a starting dose of 4.5 mg/kg/day for 12 weeks, then titrated to 6.0 mg/kg/day, or to placebo. The primary outcomes included mean change from baseline to week 24 in the ADAS-Cog and the ADCS-ADL. The secondary outcomes included MMSE, CDR, and the CIBIC-Plus scale (week 8, week 12, and week 24), and safety was assessed for each masitinib dose level. Thus, there was no bias in outcome measures. Baseline characteristics between arms were well balanced, but statistical significance was not reported. Masitinib arm (*n* = 182) displayed significant improvements in the primary endpoint of cognition measured through the ADAS-Cog, −1.46 (95% CI [−2.46, −0.45]) over the placebo (*n* = 176; 0.69 (95% CI [−0.36, 1.75]). Also, an overall improvement of functioning was recorded according to ADCS-ADL (difference between groups of 1.82 (97.5% CI [−0.15, 3.79]); *p* = 0.038), and maculo-papular rash, neutropenia, and hypoalbuminemia were the most important side effects. In the group masitinib 4.5 mg/kg/day, *n* = 5 protocol violations were reported, and *n* = 0 in the other group and discontinuation before week 24 was detected in both groups. No bias of selective reporting occurred.

The study by Tsai et al., 2019 (NCT019666666 and NCT02133846) [57] focused on the microtubule stabilizer TPI-287 in the frame of a basket-design clinical trial, allowing investigation on different clinical syndromes that had not been used for neurodegenerative diseases. This study is a two parallel-design, double-blind, placebo-controlled phase 1 randomized clinical trial enrolling patients with AD (*n* = 29), four-repeat tauopathies—i.e., progressive supranuclear palsy (*n* = 14) and β-amyloid–negative corticobasal syndrome (*n* = 30). History of significant peripheral neuropathy was the most important exclusion criterion. All the participants in the study and staff were blind to treatment assignment. Patients were randomly assigned by an unblinded pharmacist, thus raising some concern in terms of bias, in an 8:3 ratio drug to placebo in three sequential dose groups treated with 2.0, 6.3, or 20.0 mg/m^2^ of intravenous TPI-287 once every 3 weeks for 9 weeks. An optional open-label extension of 6 weeks was planned. The trial obtained ethical approval from the University of California, San Francisco (UCSF), and from the University of Alabama; informed consent was provided at screening. No bias was reported in the domain of reporting, since an independent data and safety monitoring board was included and the trial followed the Consolidated Standards of Reporting Trials (CONSORT) reporting guideline. Hence, there was no bias in the measures of outcome tools. Statistically significant differences in baseline characteristics were not reported. The primary outcome was the safety and tolerability, and the secondary outcome was the pharmacokinetic profile, whilst the cognitive assessment was included in the exploratory clinical end points, consisting of the MMSE, the ADAS-Cog and the ADCS-ADL. Three severe anaphylactoid reactions were observed in the arm treated with AD, while none occurred in patients with 4RT. Hence, the maximum tolerated dose was 6.3 mg/m^2^ for AD and 20.0 mg/m^2^ for 4RT. Patients in the treatment groups presented a higher incidence of headache, dizziness, constipation, diarrhea, and nausea. Decline in MMSE median scores was observed to be reduced (*p* = 0.04) in the treatment (0 [−4 to 4]) compared with the placebo arms (−3 [−4 to 1]). On the contrary, a worsening (*p* = 0.03) of the Clinical Dementia Rating scale sum of boxes with frontotemporal dementia was reported in the 4RT treatment arm (0.5 [−3 to 5]) compared with the placebo arm (−0.75 [−3 to 3]. Worsening was also observed for the median Geriatric Depression Screen score in the treatment group (1 [−8 to 6]) compared with the placebo (−1 [−4 to 1]). Six patients in the AD trial and two in the 4RT trial discontinued the treatment. No bias of selective reporting was detected. 

#### 3.2.3. Resveratrol

The following studies were concerned with the effectiveness of resveratrol: Turner et al., 2015 [56]; Moussa et al., 2017 [92]; Zhu et al., 2018 [93].

The study by Turner et al., 2015 [56] is a randomized, placebo-controlled, double-blind, multicenter phase 2 trial investigating the effect of resveratrol in terms of safety, biomarkers variation, brain volume, MMSE, ADCS-ADL, CDR-SB, neuropsychiatric inventory (NPI), APOE genotype, insulin, and glucose metabolism in patients aged > 49 years, affected by probable AD, based on the criteria of the National Institute of Neurological and Communicative Disorders and Stroke–Alzheimer’s Disease and Related Disorders Association, with MMSE = 14–26 at screening, modified Hachinski Score < 5, normal laboratory values, and stable medications for 4 months. Hence, no bias in the measures of outcome tools was detected. The following exclusion criteria were applied: non-AD dementia, Down syndrome, sensory impairments precluding participation, pregnancy, contraindication to lumbar puncture or MRI, more than four microhemorrhages according to a recent MRI, treated diabetes mellitus, use of resveratrol-containing supplements, or unsuitable disorders and laboratory findings. The allocation to resveratrol or placebo group consisted of a stratified by site permuted block method with an allocation ratio of 1:1 after signature of the informed consent, from the Informatics Core. However, biases occurred in the allocation domain, since the sample sizes (60 per group) were determined from power analyses, but the trial was underpowered to detect significant differences in cognitive and neuropsychiatric outcomes. The baseline characteristics of AD duration demonstrated a statistically significant (*p* < 0.001) difference between the treatment and placebo groups from year of symptom onset, therefore some concerns in terms of selection bias were raised. The most common AEs were nausea and diarrhea (in 42% of the group of treatment vs. 33% of the placebo group, *p* = 0.35). No differences were reported in participants who experienced at least one serious AE (20.3% on drug, 18.2% on placebo). After 52 weeks, the levels of CSF Aβ40 declined from 6.574 ± 2.346 to 6.513 ± 2.279 ng/mL in the treatment group and from 6.560 ± 2.190 to 5.622 ± 1.736 ng/mL in the placebo group. Brain volume loss increased with resveratrol treatment. Eight patients in the resveratrol group and seven in the placebo group discontinued the trial. No bias of selective reporting was detected. 

The study by Moussa et al., 2017 [92] is a retrospective study of a randomized, placebo-controlled, double-blind, multi-site, phase 2 trial (NCT01504854), thus raising some concerns in terms of domain 1 regarding allocation and study design. It investigated the effects of resveratrol (500 mg orally once daily, with dose escalation by 500-mg increments every 13 weeks to 1000 mg twice daily) on mild-moderate AD subjects with CSF Aβ42 < 600 ng/mL (*n* = 19 resveratrol-treated and *n* = 19 placebo-treated) for 52 weeks. Use of concurrent medications for AD (e.g., cholinesterase inhibitors) was allowed. Baseline characteristics were similar, but duration of diagnosis was longer in the placebo group. Drop-out was less than foreseen, hence preventing missing outcome data bias. No bias of selective reporting was detected. Cognitive outcomes included MMSE assessment. Hence, no bias in the measures of outcome tools was found. Due to the retrospective nature, this study does not meet with the criteria for inclusion in the graph of the risk of bias for randomized, clinical trials. Plasma biomarkers evaluation highlighted no significant differences. After 52 weeks, an ADL score of ADL-CL reduced resulted in both the resveratrol group and in the placebo group compared to the baseline (*p* < 0.001), whilst no significant change was detected in MMSE scores.

The study by Zhu et al., 2018 [93] (NCT00678431) investigated for 1 year the safety, tolerability, and efficacy of an oral preparation of low-dose resveratrol, glucose, and malate (compared with placebo; both were ingested with an 8 oz glass of commercial unsweetened grape juice twice a day) in patients with mild to moderate AD, free of life-threatening disease, and devoid of contraindications to the use of the study product on ADAS-Cog (primary endpoint), MMSE, ADCS-ADL, and NPI. Hence, no bias in the measures of outcome tools could be detected. It is a pilot study with placebo-controlled, parallel design, in which enrolled participants were randomly assigned to the treatment or placebo group, with a follow-up after 3, 6, and 12 months. N = 35 participants per group were needed to obtain 80% power to detect a difference in mean ADAS-Cog change with α = 0.05. Since *n* = 32 patients were randomized, there is some concern of risk of bias in this domain. Moreover, the outcomes were assessed by trained clinicians, blind to participants’ treatment assignment. Randomization was centrally generated and stratified by site. Baseline characteristics were not statistically different between arms. Inclusion criteria were the following: living in the community, but with supervision/a partner available for administration of study medications, to accompany the subject to all scheduled visits, and to complete informant-based assessments; subjected to stable medical condition and stable use of non-excluded medications (all apart from drugs endowed with significant central anticholinergic or antihistaminic effects, and experimental drugs); with modified Hachinski score < 4 [29]; able to complete baseline assessments in English or Spanish; and to ingest an oral agent. Exclusion criteria included: active liver or renal disease; using another investigational agent within 2 months of the screening visit; history of clinically significant stroke or of seizures, head injury with loss of consciousness, and/or immediate confusion after the injury; major psychiatric disorders, blindness, deafness, language difficulties, or other disabilities that could interfere with the assessment. There was only one early termination. No bias of selective reporting was detected. ADAS-Cog mean change scores from baseline were −0.83 ± 7.88, 1.45 ± 6.27, and 5.33 ± 14.46 at month 3, 6, and 12 for the control group, respectively, and −0.21 ± 6.57, 1.33 ± 6.10, and 2.00 ± 15.36 for the treatment group, respectively. For the secondary endpoints, MMSE mean change scores from baseline were 0.09 ± 2.39, −1.27 ± 2.65, and −3.27 ± 3.47 at month 3, 6, and 12 for the control group, respectively, and 1.15 ± 2.91, 0.45 ± 1.97, and −1.73 ± 4.43 for the treatment group, respectively. ADCS-ADL mean change scores from baseline were −1.83 ± 7.43, −1.27 ± 8.81, and −5.58 ± 11.37 at month 3, 6, and 12 for the control group, respectively, and −0.64 ± 5.47, −1.83 ± 8.31, and −0.75 ± 9.00 for the treatment group, respectively. NPI mean change scores from baseline were 4.67 ± 6.95, 2.27 ± 12.46, and 3.17 ± 10.92 at month 3, 6, and 12 for the control group, respectively, and −1.64 ± 3.73, −0.25 ± 4.94, and 0.75 ± 6.69 for the treatment group, respectively. However, the differences were not statistically significant. None of the reported adverse events were determined to be study-related. No bias of selective reporting was present. The authors concluded that a larger study is needed to determine the efficacy of low-dose resveratrol. 

#### 3.2.4. Spermidine

The study by Wirth et al., 2018 [94] and the study by Pekar et al., 2021 [95] investigated the effects of spermidine on aged patients at risk or suffering from dementia. 

The study by Wirth et al., 2018 [94] (NCT02755246) is a randomized, placebo-controlled, double-blind Phase IIa pilot trial in which the efficacy of a three-month spermidine-rich plant extract supplement (daily spermidine dose of 1.2 mg) on memory tasks (mnemonic similarity test) was assessed in comparison with a placebo. The study enrolled thirty individuals, aged 60–80 and fluent German speakers, affected by subjective cognitive decline. Neuropsychological tests were used as additional outcome measure. No bias in the measures of outcome tools was evident. Informed consent was obtained for each participant, and the study was conducted in accordance with the declaration of Helsinki. According to sample size calculation, 15 participants per group and 15 participants in the placebo group are sufficient to highlight difference with 80% power and a two-sided significance level of α = 0.049. One participant per arm dropped out. Spermidine and placebo capsules were identical in shape, color, taste, and smell. The baseline characteristics are expressed as mean, standard deviation (SD), and interquartile range (IQR), but without indication of statistically significant differences, thus raising some concern of bias in domain 1. Spermidine supplementation improved mnemonic discrimination performance, which was proven by a medium effect size (Cohen’s d and 95% CI = 0.77 [0–1.53]). On the contrary, this effect could not be reported for neuropsychological tests. No bias of selective reporting occurred. The Authors suggested that a follow-up Phase IIb randomized controlled trial is required. The later SmartAge study protocol [96] (NCT03094546) for a monocentric, randomized, double-blind, placebo-controlled Phase IIb trial aims at enhancing cognitive and brain health in older individuals with subjective cognitive decline, assessing the effects of spermidine supplementation on memory, neurocognitive, behavioral, and neuroimaging (MRI) parameters.

Furthermore, the study by Pekar et al., 2021 [95] is a three-month randomized, two-group, double-blind, multicentric, and longitudinal trial recruiting 85 subjects aged between 60 and 96 years in nursing homes, without dementia, or with mild, moderate, or severe dementia. The exclusion criteria are the following: use of antidementia medication; change of previous medication; withdrawal by choice or participation to another study. Written informed consent was achieved per each participant, in accordance with the Ethics Committee. The first group was administered a grain roll (roll A) with wheat germ containing 3.3 mg of spermidine after baking. On the other side, roll B contained 1.9 mg of spermidine. On average, the subjects ate 68 rolls during the study, with a maximum number of rolls of 79 and a minimum of zero. This raises concern in terms of the dose administered and of domains 2 and 3 regarding risk of bias. The tests to measure the endpoint scores are the following: verbal fluency; Boston naming test; MMSE; learn, recall and recognize a word list; sign and recall figures; Trail A and B; phonemic fluid. Hence, no risk of bias in the measure of the outcomes occurred, and no bias of selective reporting occurred. The group of subjects with mild dementia showed an increase of 2.23 points (*p* = 0.026) in MMSE and 1.99 (*p* = 0.47) in phonematic fluidity.

The most important features of the studies included in the analysis are reported in Table 1.

## 4. Discussion

Autophagy is a highly evolutionarily conserved process [2], undergoing derangement during aging, neurodegenerative diseases, dementia [3,4], and neuropathic pain [97,98]. There is increasing evidence for the role of the induction of autophagy on the improvement of cognitive domains of dementia [99], as supported by a network analysis that highlighted the involvement of the dysregulation of several phases of the autophagy molecular pathway in dementia in view of a systems biology approach [100]. The present systematic review highlights the paucity of homogeneous clinical trials in the field of autophagy modulation for the improvement of symptoms associated with dementia. Database searching and reference list screening permitted the retrieval of 3067 records, but after full text screening only 10 results met the criteria to be included in the analysis, not allowing meta-analysis to be performed due to their different design. 

The efficacy of metformin was investigated in studies by Koenig et al., 2017 [87] and by Luchsinger et al., 2016 [88], whilst the study by Piette et al., 2011 [37] (NCT00976118) and the study by Dubois et al., 2023 [91] (AB09004) investigated the effect of masitinib. Also, in the field of antitumoral drugs, the study by Tsai et al., 2019 (NCT019666666 and NCT02133846) [57] examined the efficacy of the microtubule stabilizer TPI-287. The effectiveness of resveratrol was evaluated by the study of Turner et al., 2015 [56], Moussa et al., 2017 [92], and Zhu et al., 2018 [93]. Finally, the study by Wirth et al., 2018 [94] and the study by Pekar et al., 2021 [95] investigated the effects of spermidine. The only retrospective study was the study by Moussa et al., 2017 [92] (NCT01504854).

Despite a common lack of serious biases, occurring mainly in the selection of non-significant baseline differences and in the sample power, the heterogeneity of intervention, patients enrolled, endpoints assessment, and measures of outcomes prevents generalization of results. The outcome measures most influenced by the treatment were MMSE and executive functioning, pointing at a role for metformin, resveratrol, and antitumoral drugs masitinib and TPI-287. The cellular and molecular effects on the multi-step process of autophagy for each compound deserves investigation. Among the mechanisms found, evidence is accumulating in favor of the role of induction of autophagy by metformin on adenosine monophosphate-activated protein kinase (AMPK)-related pathways, including inhibition of mTOR and activation of unc-51 like autophagy activating kinase 1 (ULK1), and of CCAAT enhancer-binding protein delta (CEBPD) [101]. The finding that metformin induces lysosomal-dependent chaperone-mediated autophagy through TAK1 (transforming growth factor beta-activated kinase 1)-IKK α/βHsc70 signaling is linked to a reversion the molecular and behavioral phenotypes of AD [102]. Resveratrol activates autophagy and inhibits AKT/mTOR signaling, improving cognitive function [103]. Moreover, resveratrol can significantly enhance mitophagy, increasing acidic vesicular organelle number, LC3-II/LC3-I ratio, and Parkin and Beclin-1 expression [104]. Among its several targets, masitinib can revert vascular injury, reduce radical oxygen species and mitochondria dysfunction, restoring the normal mitophagy levels, reducing the upregulation of BNIP3L/NIX, PINK1, and Parkin [105]. TPI-287 induction may occur via a reduction of the impairment of autophagy by means of microtubule stabilization since autophagosomes are mainly produced in the distal tip of the axon, thus needing microtubule integrity for retrograde transport toward the cell soma [106,107]. The overall safety profile of these drugs is tolerable. The different design of the eligible studies included the enrollment of people at risk of dementia, with probable diagnosis, with mild to moderate dementia, or with 4RT pathologies. The domain of behavioral symptoms is less investigated than cognitive improvement, thus prompting new prospective studies to assess in a comparable manner these symptoms. Furthermore, the concurrent validity of cognitive scales should be more deeply investigated, since the effects of autophagy inducers were differentially detected by the various tools used. A retrospective longitudinal study including N = 62 patients with autopsy-confirmed diagnoses of AD and dementia with Lewy bodies highlights different clinical trajectories of cognitive decline [108], pointing at the need to study the potential different effects of autophagy inducers across dementias of various etiology.

In the 1990′s, Bcl-2-associated athanogene 3 (BAG3) was first identified as a member of a family of BAG-1-related proteins [109] that is subjected to upregulation during stress conditions [110]. BAG3 can be involved in correct protein homeostasis during aging, and hence in the prevention of the development of neurodegenerative diseases [111,112]. It enhances autophagy through the promotion of glutamine consumption and glutaminolysis [113]. In particular, glutamine deprivation can decrease the phosphorylation of mTOR in the short-term, while it can activate the AKT-mTOR pathway in the long-term [114]. Chronic autophagy impairment, in which the glutamate neuron-astrocyte pathway may play a role [115,116], is implicated in protein accumulation and neurodegeneration. Therefore, based on the duration of damage, glutamine supplementation [114] and glutaminase inhibitors [116] deserve investigation, since glutamate is converted from glutamine by glutaminase and metabolized via the tricarboxylic acid cycle [117]. In fact, the modulation of glutamate to deactivate the mTOR pathway—since inactivation of mTOR can attenuate autophagy impairment also under glutamine deprivation [114]—is an aspect of the utmost importance. BAG3 is a “key player” in selective macroautophagy (different from the proteasomal system linked to BAG1), and its overexpression is suggested to increase autophagic flux and lysosomal rearrangement, which are fundamental for the response to redox stress as demonstrated in experiments using the HT22 wild-type hippocampal neuronal cell line very susceptible to oxidative stress and HT22 cells resistant to hydrogen peroxide-induced oxidative stress (OxSR cells) [118]. As previously reported, modulation of oxidative stress and of mitochondrial fission-fusion balance is thought to be involved in the mechanisms of action to alleviate dementia pathogenesis of the autophagy inducers identified in the present analysis. The essential oil of bergamot (BEO) is able to enhance both basal and induced autophagy [119], thus proving to be a promising candidate for the improvement of AD-related pathogenesis. Moreover, BEO was subjected to a step-by-step preclinical-to-clinical pathway to obtain proof-of-concept of its efficacy and safety as an engineered, nanotechnological, pharmaceutical form, known as NanoBEO, released by an airless dispenser [120,121,122,123,124,125,126,127,128]. Pain processing is subjected to an important dysfunction during aging [129,130,131], and it is involved in the development of agitation [132,133] and in the reduction of the quality of life [134] of demented patients. Furthermore, a high-throughput machine-learning approach combined with a cross-species screening platform was recently proposed to identify novel mitophagy-inducers from a natural product library to validate for efficacy [135]. 

## 5. Conclusions

The social burden of dementia is remarkable, fostering research to identify disease-modifying drugs able to counteract cognitive and behavioral symptoms, improving functioning and quality of life. The results of the present systematic review point at a role for metformin, resveratrol, masitinib and TPI-287 in outcomes including cognition and executive functioning, with an overall tolerable safety profile. However, behavioral symptoms were found to be less investigated, and the lack of homogeneous design impedes generalization of results. Moreover, future studies need to evaluate the possible correlation of imaging data with prospective longitudinal atrophy, in agreement with the finding that the global intensity and the distribution of tau-positron emission tomography (PET) can foresee the rate and the topography of subsequent atrophy [136]. These findings should prompt new studies assessing the role of nutraceuticals in neurodegenerative diseases [137] and of natural products in dementia, mainly with analgesic activity as potentially useful and safe add-on therapies [121,125], without excluding these patients from painkiller trials [138], also for migraines [139,140].

## Figures and Tables

**Figure 2:**
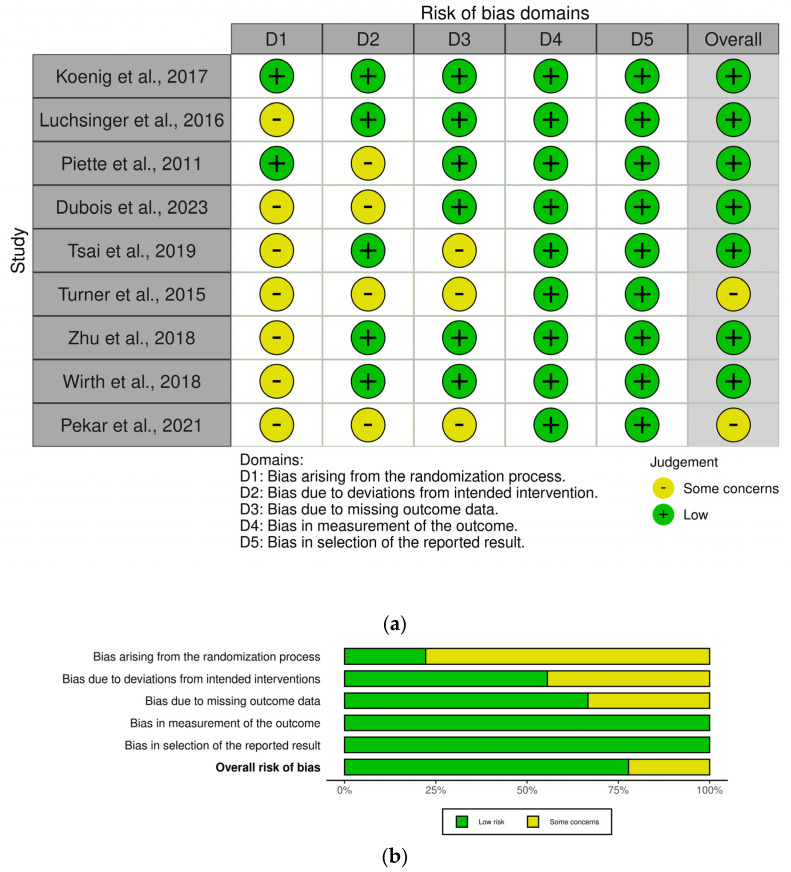
Risk of bias assessment of the studies included in the analysis [37,56,57,87,88,91,93,94,95] as traffic-light plot (**a**) and weighted bar plots (**b**). The visualization of the risk of bias assessment is produced with the Cochrane robvis visualization tool [52].

**Table 1:** Main features of the studies included in the systematic analysis.

Study	Population	Intervention	Comparator	Outcome Measures	Study Design	Results
Koenig et al., 2017 (NCT01965756) [87]	Patients aged 55–80 years, without history of diabetes or pre-diabetes, with diagnosed mild cognitive impairment (MCI) or early AD [graded as Cognitive Dementia Rating (CDR)-Global score ≤ 1.0 and Mini-Mental State Examination (MMSE) score > 19]; presenting at least one positive biomarker of AD, merging from CSF analysis, positron emission tomography (PET), or amyloid scan; baseline Geriatric Depression Scale (GDS) total < 6; modified Hachinski Ischemic Score < 4; any acetylcholinesterase inhibitors on a stable dose for at least 2 months before baseline; without any other CNS, pancreatic, liver, or renal concerns, and without history of substance abuse/dependence within the past two years; contraindication to participation in MRI or lumbar puncture; not treated with any other anticholinergic medications	Metformin	Placebo	Cognitive subscale, immediate learning and delayed recall of word list of the Alzheimer’s Disease Assessment Scale (ADAS-Cog). The computerized Cambridge Neuropsychological Test Automated Battery (CANTAB) was performed to assess learning, executive functioning (Trails-B on backwards Digit Span), attention, language, and motor speed. The assessment of depression was performed through the Geriatric Depression Scale (GDS). The Dementia Severity Rating Scale (DSRS) was used for the evaluation of functioning	Deep-phenotyping, randomized, double-blinded, placebo-controlled crossover pilot study	The only statistically significant treatment effect was reported for executive functioning (Trails-B: *p* < 0.05).
Luchsinger et al., 2016 (NCT00620191) [88]	55–90-year-old patients suffering from amnestic mild cognitive impairment (aMCI) according to Petersen criteria, without treated diabetes and overweight or obese (body mass index, BMI ≥ 25)	Metformin	Placebo	Total recall of the Bushcke Selective Reminding Test; ADAS-Cog; Alzheimer’s Disease Cooperative Study (ADCS); Clinical Global Impression of Change for Mild Cognitive Impairment (CGIC-MCI); logical memory II delayed paragraph recall sub-test of the Wechsler Memory Scale Revised (WMS-R); MMSE; Neuropsychiatric Inventory Questionnaire (NPI-Q) [49]; the digit span backwards; relative glucose uptake (rCMRgl) in the posterior cingulate-precuneous measured by non-quantitative brain ^[18]^F-labeled 2-deoxy-2-fluoro-D-glucose (FDG) positron emission tomography (PET) with magnetic resonance imaging (MRI) co-registration; plasma amyloid β42 levels; genotyping of APOE polymorphisms rs7412 and rs429358	Double-blind, placebo-controlled, randomized, pilot trial	No statistically significant differences for the memory, cognitive and neuropsychiatric outcome measures, or for the other outcomes. After adjusting for baseline ADAS-Cog, changes in total recall of the Selective Reminding Test favored the metformin group (9.7 ± 8.5 vs. 5.3 ± 8.5; *p* = 0.02)
Piette et al., 2011 [37] (NCT00976118)	Patients with mild-to-moderate Alzheimer’s disease (AD) (according to the Diagnostic and Statistical Manual Of Mental Disorders IV criteria, and to the National Institute of Neurological and Communicative Disorders and Stroke-Alzheimer’s Disease and Related Disorders Association criteria), with a baseline MMSE score between 12 and 26 and a baseline Clinical Dementia Rating (CDR) of 1 or 2	Masitinib as an adjunct to cholinesterase inhibitor and/or memantine	Placebo	The primary endpoint was the ADAS-Cog mean difference at week 24 from baseline defined by a blind Data Review Committee prior to unblinding. Secondary outcomes included the following assessments: the Alzheimer’s Disease Cooperative Study Activities of Daily Living Inventory (ADCS-ADL); the Clinician’s Interview-Based Impression of Change-plus caregiver input (CIBIC-Plus); the MMSE and the CDR; safety assessment through physical examinations, vital signs, clinical laboratory evaluations, and monitoring of adverse events	Randomized, double-blind, parallel-group, placebo-controlled, phase II study	The decline of cognitive functions according to ADAS-Cog was significantly higher in the placebo group in comparison with the masitinib group after 12 and 24 weeks (50% vs. 6% for both; *p* = 0.040 and *p* = 0.046, respectively). The mean absolute change values of MMSE were as follows: at week 12, masitinib (0.1 ± 2.5, *n* = 17) and placebo (−2.1 ± 2.5, *n* = 7), *p* = 0.047; at week 24, masitinib (−0.1 ± 4.3, *n* = 16) and placebo (−3.3 ± 3.3, *n* = 7), *p* = 0.031.
Dubois et al., 2023 [91] (AB09004)	Patients over 50 years, diagnosed with mild-to-moderate, probable AD and with MMSE = 12–25 were randomized (1:1) to receive oral masitinib 4.5 mg/kg/day, as two intakes, or placebo in the first group. In the second, independent, parallel group, patients were randomized (2:1) to masitinib at a starting dose of 4.5 mg/kg/day for 12 weeks, then titrated to 6.0 mg/kg/day, or to placebo	Masitinib	Placebo	The primary outcomes included mean change from baseline to week 24 in the ADAS-Cog and the ADCS-ADL. The secondary outcomes included MMSE, CDR, and CIBIC-Plus scale (week 8, week 12, and week 24), and safety was assessed for each dose	Randomized, double-blind, two parallel-group (four-arm), placebo-controlled trial	Masitinib arm (*n* = 182) displayed significant improvement in the primary endpoint of cognition measured through the ADAS-cog, −1.46 (95% CI [−2.46, −0.45]) over placebo (*n* = 176; 0.69 (95% CI [−0.36, 1.75]).
Tsai et al., 2019 (NCT019666666 and NCT02133846) [57]	Patients affected by AD (*n* = 29), four-repeat tauopathies—i.e., progressive supranuclear palsy (*n* = 14) and β-amyloid–negative corticobasal syndrome (*n* = 30)	TBI-287	Placebo	The primary outcome was safety and tolerability, and the secondary outcome was the pharmacokinetic profile, whilst the cognitive assessment was included in the exploratory clinical end points, consisting in the MMSE, the ADAS-Cog, and the ADCS-ADL	Basket, two parallel-design, double-blind, placebo-controlled phase 1 randomized clinical trial	Three severe anaphylactoid reactions were observed in the arm treated with AD, while none occurred in patients with 4RT. Hence, the maximum tolerated dose was 6.3 mg/m^2^ for AD and 20.0 mg/m^2^ for 4RT. Patients in the treatment groups present-ed a higher incidence of headaches, dizziness, constipation, diarrhea, and nausea. Decline in MMSE median scores was observed to be reduced (*p* = 0.04) in the treatment (0 [−4 to 4]) compared with the placebo arms (−3 [−4 to 1]). On the contrary, a worsening (*p* = 0.03) of the Clinical Dementia Rating scale sum of boxes with frontotemporal dementia measures median score was reported in the 4RT treatment arm (0.5 [−3 to 5]) compared with the placebo arm (−0.75 [−3 to 3]. Worsening was observed also for the median Geriatric Depression Screen score in the treatment group in the 4RT treatment arm (1 [−8 to 6]) compared with placebo (−1 [−4 to 1])
Turner et al., 2015 [56]	Patients aged > 49 years, affected by probable AD, based on the criteria of the National Institute of Neurological and Communicative Disorders and Stroke–Alzheimer’s Disease and Related Disorders Association, with MMSE = 14–26 at screening, modified Hachinski Score < 5, normal laboratory values and stable medications for 4 months	Resveratrol	Placebo	Safety, biomarkers variation, brain volume, MMSE, ADCS-ADL, CDR-SB, neuropsychiatric inventory (NPI), APOE genotype, insulin and glucose metabolism	Randomized, placebo-controlled, double-blind, multicenter phase 2 trial	The trial was underpowered to detect significant differences in cognitive and neuropsychiatric outcomes
Moussa et al., 2017 [92] (NCT01504854)	Patients suffering from mild-moderate AD with CSF Aβ42 < 600 ng/mL	Resveratrol	Placebo	Plasma biomarkers and cognitive outcomes including MMSE assessment	Retrospective study of a randomized, placebo-controlled, double-blind, multi-site, phase 2 trial	Plasma biomarkers evaluation highlighted no significant differences. After 52 weeks, ADL score of ADL-CL reduced both in resveratrol group and in placebo group compared to baseline (*p* < 0.001), whilst no significant change was detected in MMSE scores
Zhu et al., 2018 [93] (NCT00678431)	Patients with mild to moderate AD, free of life-threatening disease, and devoid of contraindications to the use of the study product	Low-dose resveratrol, glucose, and malate	Placebo	ADAS-Cog (primary endpoint), MMSE, ADCS-ADL, and NPI	Pilot study with placebo-controlled, parallel design	ADAS-Cog mean change scores from baseline were −0.83 ± 7.88, 1.45 ± 6.27, and 5.33 ± 14.46 at month 3, 6, and 12 for the control group, and −0.21 ± 6.57, 1.33 ± 6.10, and 2.00 ± 15.36 for the treatment group, respectively. For the secondary endpoints, MMSE mean change scores from baseline were 0.09 ± 2.39, −1.27 ± 2.65, and −3.27 ± 3.47 at month 3, 6, and 12 for the control group, and 1.15 ± 2.91, 0.45 ± 1.97, and −1.73 ± 4.43 for the treatment group, respectively. ADCS-ADL mean change scores from baseline were −1.83 ± 7.43, −1.27 ± 8.81, and −5.58 ± 11.37 at month 3, 6, and 12 for the control group, and −0.64 ± 5.47, −1.83 ± 8.31, and −0.75 ± 9.00 for the treatment group, respectively. NPI mean change scores from baseline were 4.67 ± 6.95, 2.27 ± 12.46, and 3.17 ± 10.92 at month 3, 6, and 12 for the control group, and −1.64 ± 3.73, −0.25 ± 4.94, and 0.75 ± 6.69 for the treatment group, respectively. However, the differences were not significantly different
Wirth et al., 2018 [94] (NCT02755246)	Individuals aged 60–80 and fluent German speakers, affected by subjective cognitive decline	Spermidine-rich plant extract supplement	Placebo capsules	Memory tasks. Neuropsychological tests were used as additional outcome	Randomized, placebo-controlled, double-blind Phase IIa pilot trial	Spermidine supplementation improved mnemonic discrimination performance, which was proven by a medium effect size (Cohen’s d and 95% CI = 0.77 [0, 1.53]). This effect could not be reported for neuropsychological tests
Pekar et al., 2021 [95]	Patients without dementia or with mild, moderate, or severe dementia	Roll A with wheat germ containing 3.3 mg of spermidine after baking	Roll B contained 1.9 mg of spermidine	Verbal fluency; Boston naming test; MMSE; learn, recall and recognize a word list; sign and recall figures; Trail A and B; phonemic fluid	Randomized, two-group, double-blind, multicentric, and longitudinal trial	The group of subjects with mild dementia showed an increase of 2.23 points (*p* = 0.026) in MMSE and 1.99 (*p* = 0.47) in phonematic fluidity

The assessment of the risk of bias is reported in Figure 2.

## Data Availability

Data is contained within the article.

## References

[1] (2022). Estimation of the global prevalence of dementia in 2019 and forecasted prevalence in 2050: An analysis for the Global Burden of Disease Study 2019. Lancet Public Health.

[2] Harding T.M., Morano K.A., Scott S.V., Klionsky D.J. (1995). Isolation and characterization of yeast mutants in the cytoplasm to vacuole protein targeting pathway. J. Cell Biol..

[3] Rubinsztein D.C., Mariño G., Kroemer G. (2011). Autophagy and aging. Cell.

[4] Cuervo A.M. (2008). Autophagy and aging: Keeping that old broom working. Trends Genet. TIG.

[5] Chu C.T. (2006). Autophagic Stress in Neuronal Injury and Disease. J. Neuropathol. Exp. Neurol..

[6] Metaxakis A., Ploumi C., Tavernarakis N. (2018). Autophagy in Age-Associated Neurodegeneration. Cells.

[7] Nixon R.A., Wegiel J., Kumar A., Yu W.H., Peterhoff C., Cataldo A., Cuervo A.M. (2005). Extensive Involvement of Autophagy in Alzheimer Disease: An Immuno-Electron Microscopy Study. J. Neuropathol. Exp. Neurol..

[8] Grbovic O.M., Mathews P.M., Jiang Y., Schmidt S.D., Dinakar R., Summers-Terio N.B., Ceresa B.P., Nixon R.A., Cataldo A.M. (2003). Rab5-stimulated up-regulation of the endocytic pathway increases intracellular beta-cleaved amyloid precursor protein carboxyl-terminal fragment levels and Abeta production. J. Biol. Chem..

[9] Cataldo A.M., Barnett J.L., Berman S.A., Li J., Quarless S., Bursztajn S., Lippa C., Nixon R.A. (1995). Gene expression and cellular content of cathepsin D in Alzheimer’s disease brain: Evidence for early up-regulation of the endosomal-lysosomal system. Neuron.

[10] Petiot A., Pattingre S., Arico S., Meley D., Codogno P. (2002). Diversity of signaling controls of macroautophagy in mammalian cells. Cell Struct. Funct..

[11] Yu W.H., Cuervo A.M., Kumar A., Peterhoff C.M., Schmidt S.D., Lee J.-H., Mohan P.S., Mercken M., Farmery M.R., Tjernberg L.O. (2005). Macroautophagy—A novel β-amyloid peptide-generating pathway activated in Alzheimer’s disease. J. Cell Biol..

[12] Chen G.F., Xu T.H., Yan Y., Zhou Y.R., Jiang Y., Melcher K., Xu H.E. (2017). Amyloid beta: Structure, biology and structure-based therapeutic development. Acta Pharmacol. Sin..

[13] Mizushima N., Levine B., Cuervo A.M., Klionsky D.J. (2008). Autophagy fights disease through cellular self-digestion. Nature.

[14] Cataldo A.M., Barnett J.L., Pieroni C., Nixon R.A. (1997). Increased neuronal endocytosis and protease delivery to early endosomes in sporadic Alzheimer’s disease: Neuropathologic evidence for a mechanism of increased beta-amyloidogenesis. J. Neurosci. Off. J. Soc. Neurosci..

[15] Orr M.E., Oddo S. (2013). Autophagic/lysosomal dysfunction in Alzheimer’s disease. Alzheimers Res. Ther..

[16] Di Meco A., Curtis M.E., Lauretti E., Praticò D. (2020). Autophagy Dysfunction in Alzheimer’s Disease: Mechanistic Insights and New Therapeutic Opportunities. Biol. Psychiatry.

[17] Lee V.M., Goedert M., Trojanowski J.Q. (2001). Neurodegenerative tauopathies. Annu. Rev. Neurosci..

[18] Ling D., Magallanes M., Salvaterra P.M. (2014). Accumulation of amyloid-like Aβ_1–42_ in AEL (autophagy-endosomal-lysosomal) vesicles: Potential implications for plaque biogenesis. ASN Neuro.

[19] Geng P., Zhang J., Dai W., Han X., Tan Q., Cheng D., Fang P., Liu X. (2018). Autophagic Degradation Deficit Involved in Sevoflurane-Induced Amyloid Pathology and Spatial Learning Impairment in APP/PS1 Transgenic Mice. Front. Cell. Neurosci..

[20] Rodriguez S., Hug C., Todorov P., Moret N., Boswell S.A., Evans K., Zhou G., Johnson N.T., Hyman B.T., Sorger P.K. (2021). Machine learning identifies candidates for drug repurposing in Alzheimer’s disease. Nat. Commun..

[21] Eshraghi M., Ahmadi M., Afshar S., Lorzadeh S., Adlimoghaddam A., Rezvani Jalal N., West R., Dastghaib S., Igder S., Torshizi S.R.N. (2022). Enhancing autophagy in Alzheimer’s disease through drug repositioning. Pharmacol. Ther..

[22] Ballard C., Aarsland D., Cummings J., O’Brien J., Mills R., Molinuevo J.L., Fladby T., Williams G., Doherty P., Corbett A. (2020). Drug repositioning and repurposing for Alzheimer disease. Nat. Rev. Neurol..

[23] Santana S., Recuero M., Bullido M.J., Valdivieso F., Aldudo J. (2012). Herpes simplex virus type I induces the accumulation of intracellular β-amyloid in autophagic compartments and the inhibition of the non-amyloidogenic pathway in human neuroblastoma cells. Neurobiol. Aging.

[24] Querfurth H., Lee H.K. (2021). Mammalian/mechanistic target of rapamycin (mTOR) complexes in neurodegeneration. Mol. Neurodegener..

[25] Spilman P., Podlutskaya N., Hart M.J., Debnath J., Gorostiza O., Bredesen D., Richardson A., Strong R., Galvan V. (2010). Inhibition of mTOR by rapamycin abolishes cognitive deficits and reduces amyloid-beta levels in a mouse model of Alzheimer’s disease. PLoS ONE.

[26] Boland B., Kumar A., Lee S., Platt F.M., Wegiel J., Yu W.H., Nixon R.A. (2008). Autophagy induction and autophagosome clearance in neurons: Relationship to autophagic pathology in Alzheimer’s disease. J. Neurosci. Off. J. Soc. Neurosci..

[27] Pierce A., Podlutskaya N., Halloran J.J., Hussong S.A., Lin P.Y., Burbank R., Hart M.J., Galvan V. (2013). Over-expression of heat shock factor 1 phenocopies the effect of chronic inhibition of TOR by rapamycin and is sufficient to ameliorate Alzheimer’s-like deficits in mice modeling the disease. J. Neurochem..

[28] Kickstein E., Krauss S., Thornhill P., Rutschow D., Zeller R., Sharkey J., Williamson R., Fuchs M., Köhler A., Glossmann H. (2010). Biguanide metformin acts on tau phosphorylation via mTOR/protein phosphatase 2A (PP2A) signaling. Proc. Natl. Acad. Sci. USA.

[29] Liu Y., Su Y., Wang J., Sun S., Wang T., Qiao X., Run X., Li H., Liang Z. (2013). Rapamycin decreases tau phosphorylation at Ser214 through regulation of cAMP-dependent kinase. Neurochem. Int..

[30] Frederick C., Ando K., Leroy K., Héraud C., Suain V., Buée L., Brion J.P. (2015). Rapamycin ester analog CCI-779/Temsirolimus alleviates tau pathology and improves motor deficit in mutant tau transgenic mice. J. Alzheimer’s Dis..

[31] Li L., Zhang S., Zhang X., Li T., Tang Y., Liu H., Yang W., Le W. (2013). Autophagy enhancer carbamazepine alleviates memory deficits and cerebral amyloid-β pathology in a mouse model of Alzheimer’s disease. Curr. Alzheimer Res..

[32] Bellozi P.M.Q., Lima I.V.A., Dória J.G., Vieira É.L.M., Campos A.C., Candelario-Jalil E., Reis H.J., Teixeira A.L., Ribeiro F.M., de Oliveira A.C.P. (2016). Neuroprotective effects of the anticancer drug NVP-BEZ235 (dactolisib) on amyloid-β 1–42 induced neurotoxicity and memory impairment. Sci. Rep..

[33] Bellozi P.M.Q., Gomes G.F., De Oliveira L.R., Olmo I.G., Vieira É.L.M., Ribeiro F.M., Fiebich B.L., De Oliveira A.C.P. (2019). NVP-BEZ235 (dactolisib) has protective effects in a transgenic mouse model of Alzheimer’s disease. Front. Pharmacol..

[34] Vingtdeux V., Giliberto L., Zhao H., Chandakkar P., Wu Q., Simon J.E., Janle E.M., Lobo J., Ferruzzi M.G., Davies P. (2010). AMP-activated protein kinase signaling activation by resveratrol modulates amyloid-beta peptide metabolism. J. Biol. Chem..

[35] Marambaud P., Zhao H., Davies P. (2005). Resveratrol promotes clearance of Alzheimer’s disease amyloid-beta peptides. J. Biol. Chem..

[36] Vingtdeux V., Chandakkar P., Zhao H., d’Abramo C., Davies P., Marambaud P. (2011). Novel synthetic small-molecule activators of AMPK as enhancers of autophagy and amyloid-β peptide degradation. FASEB J. Off. Publ. Fed. Am. Soc. Exp. Biol..

[37] Piette F., Belmin J., Vincent H., Schmidt N., Pariel S., Verny M., Marquis C., Mely J., Hugonot-Diener L., Kinet J.P. (2011). Masitinib as an adjunct therapy for mild-to-moderate Alzheimer’s disease: A randomised, placebo-controlled phase 2 trial. Alzheimers Res. Ther..

[38] Fitzgerald D.P., Emerson D.L., Qian Y., Anwar T., Liewehr D.J., Steinberg S.M., Silberman S., Palmieri D., Steeg P.S. (2012). TPI-287, a new taxane family member, reduces the brain metastatic colonization of breast cancer cells. Mol. Cancer Ther..

[39] Luo R., Su L.-Y., Li G., Yang J., Liu Q., Yang L.-X., Zhang D.-F., Zhou H., Xu M., Fan Y. (2020). Activation of PPARA-mediated autophagy reduces Alzheimer disease-like pathology and cognitive decline in a murine model. Autophagy.

[40] Huang W., Li Z., Zhao L., Zhao W. (2017). Simvastatin ameliorate memory deficits and inflammation in clinical and mouse model of Alzheimer’s disease via modulating the expression of miR-106b. Biomed. Pharmacother..

[41] Pinyopornpanish K., Soontornpun A., Wongpakaran T., Wongpakaran N., Tanprawate S., Pinyopornpanish K., Nadsasarn A., Pinyopornpanish M. (2022). Impact of behavioral and psychological symptoms of Alzheimer’s disease on caregiver outcomes. Sci. Rep..

[42] Vermeiren Y., Le Bastard N., Van Hemelrijck A., Drinkenburg W.H., Engelborghs S., De Deyn P.P. (2013). Behavioral correlates of cerebrospinal fluid amino acid and biogenic amine neurotransmitter alterations in dementia. Alzheimer’s Dement. J. Alzheimer’s Assoc..

[43] Wang W.W., Han R., He H.J., Wang Z., Luan X.Q., Li J., Feng L., Chen S.Y., Aman Y., Xie C.L. (2021). Delineating the Role of Mitophagy Inducers for Alzheimer Disease Patients. Aging Dis..

[44] Page M.J., McKenzie J.E., Bossuyt P.M., Boutron I., Hoffmann T.C., Mulrow C.D., Shamseer L., Tetzlaff J.M., Akl E.A., Brennan S.E. (2021). The PRISMA 2020 statement: An updated guideline for reporting systematic reviews. BMJ.

[45] Page M.J., Moher D., Bossuyt P.M., Boutron I., Hoffmann T.C., Mulrow C.D., Shamseer L., Tetzlaff J.M., Akl E.A., Brennan S.E. (2021). PRISMA 2020 explanation and elaboration: Updated guidance and exemplars for reporting systematic reviews. BMJ.

[46] Lefebvre C., Glanville J., Briscoe S., Littlewood A., Marshall C., Metzendorf M.I., Noel-Storr A., Rader T., Shokraneh F., Thomas J. (2019). Searching for and selecting studies. Cochrane Handbook for Systematic Reviews of Interventions.

[47] McGowan J., Sampson M., Salzwedel D.M., Cogo E., Foerster V., Lefebvre C. (2016). PRESS Peer Review of Electronic Search Strategies: 2015 Guideline Statement. J. Clin. Epidemiol..

[48] Ryan R., Cochrane Consumers and Communication Review Group Cochrane Consumers and Communication Review Group: Data Synthesis and Analysis. http://cccrg.cochrane.org.

[49] Hultcrantz M., Rind D., Akl E.A., Treweek S., Mustafa R.A., Iorio A., Alper B.S., Meerpohl J.J., Murad M.H., Ansari M.T. (2017). The GRADE Working Group clarifies the construct of certainty of evidence. J. Clin. Epidemiol..

[50] Sterne J.A.C., Savović J., Page M.J., Elbers R.G., Blencowe N.S., Boutron I., Cates C.J., Cheng H.-Y., Corbett M.S., Eldridge S.M. (2019). RoB 2: A revised tool for assessing risk of bias in randomised trials. BMJ.

[51] Sterne J.A., Hernán M.A., Reeves B.C., Savović J., Berkman N.D., Viswanathan M., Henry D., Altman D.G., Ansari M.T., Boutron I. (2016). ROBINS-I: A tool for assessing risk of bias in non-randomised studies of interventions. BMJ.

[52] McGuinness L.A., Higgins J.P.T. (2021). Risk-of-bias VISualization (robvis): An R package and Shiny web app for visualizing risk-of-bias assessments. Res. Synth. Methods.

[53] DerSimonian R., Kacker R. (2007). Random-effects model for meta-analysis of clinical trials: An update. Contemp. Clin. Trials.

[54] Higgins J.P.T., Thompson S.G. (2002). Quantifying heterogeneity in a meta-analysis. Stat. Med..

[55] Sterne J.A., Egger M. (2001). Funnel plots for detecting bias in meta-analysis: Guidelines on choice of axis. J. Clin. Epidemiol..

[56] Turner R.S., Ronald G.T., Suzanne C., Christopher H.v.D., Jacobo M., Brigid A.R., James B.B., Robert A.R., Rema R., Paul S.A. (2015). A randomized, double-blind, placebo-controlled trial of resveratrol for Alzheimer disease. Neurology.

[57] Tsai R.M., Miller Z., Koestler M., Rojas J.C., Ljubenkov P.A., Rosen H.J., Rabinovici G.D., Fagan A.M., Cobigo Y., Brown J.A. (2020). Reactions to Multiple Ascending Doses of the Microtubule Stabilizer TPI-287 in Patients With Alzheimer Disease, Progressive Supranuclear Palsy, and Corticobasal Syndrome: A Randomized Clinical Trial. JAMA Neurol..

[58] Gilad G.M., Gilad V.H., Wyatt R.J. (1993). Accumulation of exogenous polyamines in gerbil brain after ischemia. Mol. Chem. Neuropathol..

[59] Glantz L., Nates J.L., Trembovler V., Bass R., Shohami E. (1996). Polyamines induce blood-brain barrier disruption and edema formation in the rat. J. Basic Clin. Physiol. Pharmacol..

[60] Shin W.W., Fong W.F., Pang S.F., Wong P.C. (1985). Limited blood-brain barrier transport of polyamines. J. Neurochem..

[61] Yang Y., Chen S., Zhang Y., Lin X., Song Y., Xue Z., Qian H., Wang S., Wan G., Zheng X. (2017). Induction of autophagy by spermidine is neuroprotective via inhibition of caspase 3-mediated Beclin 1 cleavage. Cell Death Dis..

[62] European Medicines Agency 13 September 2011 EMA/HMPC/56155/2011 Committee on Herbal Medicinal Products [HMPC] [(accessed on 20 June 2022)]. https://www.ema.europa.eu/en/documents/committee-report/committee-herbal-medicinal-products-hmpc-meeting-report-12-13-september-2011_en.pdf.

[63] Wang L., Huang W., Zhan J. (2019). Grape Seed Proanthocyanidins Induce Autophagy and Modulate Survivin in HepG2 Cells and Inhibit Xenograft Tumor Growth in Vivo. Nutrients.

[64] Song C., Gao C., Wang Z. (2022). Grape-Seed-Derived Procyanidin Attenuates Chemotherapy-Induced Cognitive Impairment by Suppressing MMP-9 Activity and Related Blood-Brain-Barrier Damage. Brain Sci..

[65] Lee J., Torosyan N., Silverman D.H. (2017). Examining the impact of grape consumption on brain metabolism and cognitive function in patients with mild decline in cognition: A double-blinded placebo controlled pilot study. Exp. Gerontol..

[66] Cao G., Gong T., Du Y., Wang Y., Ge T., Liu J. (2022). Mechanism of metformin regulation in central nervous system: Progression and future perspectives. Biomed. Pharmacother..

[67] Grommes C., Karlo J.C., Caprariello A., Blankenship D., Dechant A., Landreth G.E. (2013). The PPARγ agonist pioglitazone crosses the blood-brain barrier and reduces tumor growth in a human xenograft model. Cancer Chemother. Pharmacol..

[68] Jojo G.M., Kuppusamy G. (2019). Scope of new formulation approaches in the repurposing of pioglitazone for the management of Alzheimer’s disease. J. Clin. Pharm. Ther..

[69] Watson G.S., Cholerton B.A., Reger M.A., Baker L.D., Plymate S.R., Asthana S., Fishel M.A., Kulstad J.J., Green P.S., Cook D.G. (2005). Preserved cognition in patients with early Alzheimer disease and amnestic mild cognitive impairment during treatment with rosiglitazone: A preliminary study. Am. J. Geriatr. Psychiatry Off. J. Am. Assoc. Geriatr. Psychiatry.

[70] Brodbeck J., Balestra M.E., Saunders A.M., Roses A.D., Mahley R.W., Huang Y. (2008). Rosiglitazone increases dendritic spine density and rescues spine loss caused by apolipoprotein E4 in primary cortical neurons. Proc. Natl. Acad. Sci. USA.

[71] Festuccia W.T., Oztezcan S., Laplante M., Berthiaume M., Michel C., Dohgu S., Denis R.G., Brito M.N., Brito N.A., Miller D.S. (2008). Peroxisome proliferator-activated receptor-gamma-mediated positive energy balance in the rat is associated with reduced sympathetic drive to adipose tissues and thyroid status. Endocrinology.

[72] Galindo D.C., Banks W.A., Rhea E.M. (2020). The impact of acute rosiglitazone on insulin pharmacokinetics at the blood-brain barrier. Endocrinol. Diabetes Metab..

[73] McClean P.L., Hölscher C. (2014). Liraglutide can reverse memory impairment, synaptic loss and reduce plaque load in aged APP/PS1 mice, a model of Alzheimer’s disease. Neuropharmacology.

[74] Panagaki T., Michael M., Hölscher C. (2017). Liraglutide restores chronic ER stress, autophagy impairments and apoptotic signalling in SH-SY5Y cells. Sci. Rep..

[75] He Y., Ao N., Yang J., Wang X., Jin S., Du J. (2020). The preventive effect of liraglutide on the lipotoxic liver injury via increasing autophagy. Ann. Hepatol..

[76] Tadokoro K., Morihara R., Ohta Y., Hishikawa N., Kawano S., Sasaki R., Matsumoto N., Nomura E., Nakano Y., Takahashi Y. (2019). Clinical Benefits of Antioxidative Supplement Twendee X for Mild Cognitive Impairment: A Multicenter, Randomized, Double-Blind, and Placebo-Controlled Prospective Interventional Study. J. Alzheimer’s Dis..

[77] Fukui K., You F., Kato Y., Kimura M., Harakawa Y., Yoshikawa T., Inufusa H. (2023). Twendee X, a mixed antioxidant supplement, improves cognitive function, coordination, and neurotrophic factor expression in long-term vitamin E-deficient mice. J. Clin. Biochem. Nutr..

[78] Cardoso B.R., Roberts B.R., Malpas C.B., Vivash L., Genc S., Saling M.M., Desmond P., Steward C., Hicks R.J., Callahan J. (2019). Supranutritional Sodium Selenate Supplementation Delivers Selenium to the Central Nervous System: Results from a Randomized Controlled Pilot Trial in Alzheimer’s Disease. Neurother. J. Am. Soc. Exp. NeuroTherapeutics.

[79] Malpas C.B., Vivash L., Genc S., Saling M.M., Desmond P., Steward C., Hicks R.J., Callahan J., Brodtmann A., Collins S. (2016). A Phase IIa Randomized Control Trial of VEL015 (Sodium Selenate) in Mild-Moderate Alzheimer’s Disease. J. Alzheimer’s Dis..

[80] Phelan M.J., Mulnard R.A., Gillen D.L., Schreiber S.S. (2017). Phase II Clinical Trial of Nicotinamide for the Treatment of Mild to Moderate Alzheimer’s Disease. J. Geriatr. Med. Gerontol..

[81] Rainer M., Kraxberger E., Haushofer M., Mucke H.A., Jellinger K.A. (2000). No evidence for cognitive improvement from oral nicotinamide adenine dinucleotide (NADH) in dementia. J. Neural Transm..

[82] Demarin V., Podobnik S.S., Storga-Tomic D., Kay G. (2004). Treatment of Alzheimer’s disease with stabilized oral nicotinamide adenine dinucleotide: A randomized, double-blind study. Drugs Exp. Clin. Res..

[83] Fang X., Zhang J., Zhao J., Wang L. (2022). Effect of Resveratrol Combined with Donepezil Hydrochloride on Inflammatory Factor Level and Cognitive Function Level of Patients with Alzheimer’s Disease. J. Healthc. Eng..

[84] Journal of Healthcare Engineering (2023). Retracted: Effect of Resveratrol Combined with Donepezil Hydrochloride on Inflammatory Factor Level and Cognitive Function Level of Patients with Alzheimer’s Disease. J. Healthc. Eng..

[85] Egefjord L., Gejl M., Møller A., Brændgaard H., Gottrup H., Antropova O., Møller N., Poulsen H.E., Gjedde A., Brock B. (2012). Effects of liraglutide on neurodegeneration, blood flow and cognition in Alzheimer’s disease—Protocol for a controlled, randomized double-blinded trial. Dan. Med. J..

[86] Haddaway N.R., Page M.J., Pritchard C.C., McGuinness L.A. (2022). PRISMA2020: An R package and Shiny app for producing PRISMA 2020-compliant flow diagrams, with interactivity for optimised digital transparency and Open Synthesis. Campbell Syst. Rev..

[87] Koenig A.M., Mechanic-Hamilton D., Xie S.X., Combs M.F., Cappola A.R., Xie L., Detre J.A., Wolk D.A., Arnold S.E. (2017). Effects of the Insulin Sensitizer Metformin in Alzheimer Disease: Pilot Data From a Randomized Placebo-controlled Crossover Study. Alzheimer Dis. Assoc. Disord..

[88] Luchsinger J.A., Perez T., Chang H., Mehta P., Steffener J., Pradabhan G., Ichise M., Manly J., Devanand D.P., Bagiella E. (2016). Metformin in Amnestic Mild Cognitive Impairment: Results of a Pilot Randomized Placebo Controlled Clinical Trial. J. Alzheimer’s Dis..

[89] Petersen R.C., Roberts R.O., Knopman D.S., Boeve B.F., Geda Y.E., Ivnik R.J., Smith G.E., Jack C.R. (2009). Mild cognitive impairment: Ten years later. Arch. Neurol..

[90] Petersen R.C. (2004). Mild cognitive impairment as a diagnostic entity. J. Intern. Med..

[91] Dubois B., López-Arrieta J., Lipschitz S., Doskas T., Spiru L., Moroz S., Venger O., Vermersch P., Moussy A., Mansfield C.D. (2023). Masitinib for mild-to-moderate Alzheimer’s disease: Results from a randomized, placebo-controlled, phase 3, clinical trial. Alzheimer’s Res. Ther..

[92] Moussa C., Hebron M., Huang X., Ahn J., Rissman R.A., Aisen P.S., Turner R.S. (2017). Resveratrol regulates neuro-inflammation and induces adaptive immunity in Alzheimer’s disease. J. Neuroinflammation.

[93] Zhu C.W., Grossman H., Neugroschl J., Parker S., Burden A., Luo X., Sano M. (2018). A randomized, double-blind, placebo-controlled trial of resveratrol with glucose and malate (RGM) to slow the progression of Alzheimer’s disease: A pilot study. Alzheimer’s Dement. Transl. Res. Clin. Interv..

[94] Wirth M., Benson G., Schwarz C., Köbe T., Grittner U., Schmitz D., Sigrist S.J., Bohlken J., Stekovic S., Madeo F. (2018). The effect of spermidine on memory performance in older adults at risk for dementia: A randomized controlled trial. Cortex.

[95] Pekar T., Bruckner K., Pauschenwein-Frantsich S., Gschaider A., Oppliger M., Willesberger J., Ungersbäck P., Wendzel A., Kremer A., Flak W. (2021). The positive effect of spermidine in older adults suffering from dementia. Wien. Klin. Wochenschr..

[96] Wirth M., Schwarz C., Benson G., Horn N., Buchert R., Lange C., Köbe T., Hetzer S., Maglione M., Michael E. (2019). Effects of spermidine supplementation on cognition and biomarkers in older adults with subjective cognitive decline (SmartAge)-study protocol for a randomized controlled trial. Alzheimers Res. Ther..

[97] Berliocchi L., Russo R., Maiarù M., Levato A., Bagetta G., Corasaniti M.T. (2011). Autophagy impairment in a mouse model of neuropathic pain. Mol. Pain.

[98] Yang L., Gao X., Tian D., Yang W., Xue S., Cao Z., Sun T. (2023). Resolvin D2 activates anti-inflammatory microglia via restoring autophagy flux and alleviate neuropathic pain following spinal cord injury in rats. Exp. Neurol..

[99] Djajadikerta A., Keshri S., Pavel M., Prestil R., Ryan L., Rubinsztein D.C. (2020). Autophagy Induction as a Therapeutic Strategy for Neurodegenerative Diseases. J. Mol. Biol..

[100] Caberlotto L., Nguyen T.P. (2014). A systems biology investigation of neurodegenerative dementia reveals a pivotal role of autophagy. BMC Syst. Biol..

[101] Lu G., Wu Z., Shang J., Xie Z., Chen C., Zhang C. (2021). The effects of metformin on autophagy. Biomed. Pharmacother..

[102] Xu X., Sun Y., Cen X., Shan B., Zhao Q., Xie T., Wang Z., Hou T., Xue Y., Zhang M. (2021). Metformin activates chaperone-mediated autophagy and improves disease pathologies in an Alzheimer disease mouse model. Protein Cell.

[103] Wang N., He J., Pan C., Wang J., Ma M., Shi X., Xu Z. (2019). Resveratrol Activates Autophagy via the AKT/mTOR Signaling Pathway to Improve Cognitive Dysfunction in Rats With Chronic Cerebral Hypoperfusion. Front. Neurosci..

[104] Wang H., Jiang T., Li W., Gao N., Zhang T. (2018). Resveratrol attenuates oxidative damage through activating mitophagy in an in vitro model of Alzheimer’s disease. Toxicol. Lett..

[105] An X., Ma X., Liu H., Song J., Wei T., Zhang R., Zhan X., Li H., Zhou J. (2023). Inhibition of PDGFRβ alleviates endothelial cell apoptotic injury caused by DRP-1 overexpression and mitochondria fusion failure after mitophagy. Cell Death Dis..

[106] Kirchenwitz M., Stahnke S., Grunau K., Melcher L., van Ham M., Rottner K., Steffen A., Stradal T.E.B. (2022). The autophagy inducer SMER28 attenuates microtubule dynamics mediating neuroprotection. Sci. Rep..

[107] Sferra A., Nicita F., Bertini E. (2020). Microtubule Dysfunction: A Common Feature of Neurodegenerative Diseases. Int. J. Mol. Sci..

[108] Gu Y., Kociolek A., Fernandez K.K., Cosentino S.A., Zhu C.W., Jin Z., Leverenz J.B., Stern Y.B. (2022). Clinical Trajectories at the End of Life in Autopsy-Confirmed Dementia Patients with Alzheimer Disease and Lewy Bodies Pathologies. Neurology.

[109] Takayama S., Xie Z., Reed J.C. (1999). An evolutionarily conserved family of Hsp70/Hsc70 molecular chaperone regulators. J. Biol. Chem..

[110] Doong H., Price J., Kim Y.S., Gasbarre C., Probst J., Liotta L.A., Blanchette J., Rizzo K., Kohn E. (2000). CAIR-1/BAG-3 forms an EGF-regulated ternary complex with phospholipase C-gamma and Hsp70/Hsc70. Oncogene.

[111] Tang M., Ji C., Pallo S., Rahman I., Johnson G.V.W. (2018). Nrf2 mediates the expression of BAG3 and autophagy cargo adaptor proteins and tau clearance in an age-dependent manner. Neurobiol. Aging.

[112] Lin H., Koren S.A., Cvetojevic G., Girardi P., Johnson G.V.W. (2022). The role of BAG3 in health and disease: A “Magic BAG of Tricks”. J. Cell. Biochem..

[113] Zhao S., Wang J.-M., Yan J., Zhang D.-L., Liu B.-Q., Jiang J.-Y., Li C., Li S., Meng X.-N., Wang H.-Q. (2019). BAG3 promotes autophagy and glutaminolysis via stabilizing glutaminase. Cell Death Dis..

[114] Zhou J., Chen H., Du J., Tai H., Han X., Huang N., Wang X., Gong H., Yang M., Xiao H. (2022). Glutamine Availability Regulates the Development of Aging Mediated by mTOR Signaling and Autophagy. Front. Pharmacol..

[115] Sonnewald U., Schousboe A., Schousboe A., Sonnewald U. (2016). Introduction to the Glutamate–Glutamine Cycle. The Glutamate/GABA-Glutamine Cycle: Amino Acid Neurotransmitter Homeostasis.

[116] Yuan S., Zhang Z.-W., Li Z.-L. (2017). Cell Death-Autophagy Loop and Glutamate-Glutamine Cycle in Amyotrophic Lateral Sclerosis. Front. Mol. Neurosci..

[117] McKenna M.C., Sonnewald U., Huang X., Stevenson J., Zielke H.R. (1996). Exogenous Glutamate Concentration Regulates the Metabolic Fate of Glutamate in Astrocytes. J. Neurochem..

[118] Chakraborty D., Felzen V., Hiebel C., Stürner E., Perumal N., Manicam C., Sehn E., Grus F., Wolfrum U., Behl C. (2019). Enhanced autophagic-lysosomal activity and increased BAG3-mediated selective macroautophagy as adaptive response of neuronal cells to chronic oxidative stress. Redox Biol..

[119] Russo R., Cassiano M.G., Ciociaro A., Adornetto A., Varano G.P., Chiappini C., Berliocchi L., Tassorelli C., Bagetta G., Corasaniti M.T. (2014). Role of D-Limonene in autophagy induced by bergamot essential oil in SH-SY5Y neuroblastoma cells. PLoS ONE.

[120] Rombolà L., Scuteri D., Watanabe C., Sakurada S., Hamamura K., Sakurada T., Tonin P., Corasaniti M.T., Bagetta G., Morrone L.A. (2020). Role of 5-HT1A Receptor in the Anxiolytic-Relaxant Effects of Bergamot Essential Oil in Rodent. Int. J. Mol. Sci..

[121] Scuteri D., Cassano R., Trombino S., Russo R., Mizoguchi H., Watanabe C., Hamamura K., Katsuyama S., Komatsu T., Morrone L.A. (2021). Development and Translation of NanoBEO, a Nanotechnology-Based Delivery System of Bergamot Essential Oil Deprived of Furocumarins, in the Control of Agitation in Severe Dementia. Pharmaceutics.

[122] Scuteri D., Hamamura K., Sakurada T., Watanabe C., Sakurada S., Morrone L.A., Rombolà L., Tonin P., Bagetta G., Corasaniti M.T. (2021). Efficacy of Essential Oils in Pain: A Systematic Review and Meta-Analysis of Preclinical Evidence. Front. Pharmacol..

[123] Scuteri D., Sandrini G., Tamburin S., Corasaniti M.T., Nicotera P., Tonin P., Bagetta G. (2021). Bergamot rehabilitation AgaINst agitation in dementia (BRAINAID): Study protocol for a randomized, double-blind, placebo-controlled trial to assess the efficacy of furocoumarin-free bergamot loaded in a nanotechnology-based delivery system of the essential oil in the treatment of agitation in elderly affected by severe dementia. Phytother. Res. PTR.

[124] Scuteri D., Rombolà L., Crudo M., Watanabe C., Mizoguchi H., Sakurada S., Hamamura K., Sakurada T., Morrone L.A., Tonin P. (2022). Translational Value of the Transdermal Administration of Bergamot Essential Oil and of Its Fractions. Pharmaceutics.

[125] Scuteri D., Rombolà L., Crudo M., Watanabe C., Mizoguchi H., Sakurada S., Hamamura K., Sakurada T., Tonin P., Corasaniti M.T. (2022). Preclinical Characterization of Antinociceptive Effect of Bergamot Essential Oil and of Its Fractions for Rational Translation in Complementary Therapy. Pharmaceutics.

[126] Hamamura K., Katsuyama S., Komatsu T., Scuteri D., Bagetta G., Aritake K., Sakurada T. (2020). Behavioral Effects of Continuously Administered Bergamot Essential Oil on Mice With Partial Sciatic Nerve Ligation. Front. Pharmacol..

[127] Scuteri D., Crudo M., Rombolà L., Watanabe C., Mizoguchi H., Sakurada S., Sakurada T., Greco R., Corasaniti M.T., Morrone L.A. (2018). Antinociceptive effect of inhalation of the essential oil of bergamot in mice. Fitoterapia.

[128] Scuteri D., Rombolà L., Hayashi T., Watanabe C., Sakurada S., Hamamura K., Sakurada T., Tonin P., Bagetta G., Morrone L.A. (2022). Analgesic Characteristics of NanoBEO Released by an Airless Dispenser for the Control of Agitation in Severe Dementia. Molecules.

[129] Hamm R.J., Knisely J.S. (1985). Environmentally induced analgesia: An age-related decline in an endogenous opioid system. J. Gerontol..

[130] Jourdan D., Boghossian S., Alloui A., Veyrat-Durebex C., Coudore M.A., Eschalier A., Alliot J. (2000). Age-related changes in nociception and effect of morphine in the Lou rat. Eur. J. Pain.

[131] Jourdan D., Pickering G., Marchand F., Gaulier J.M., Alliot J., Eschalier A. (2002). Impact of ageing on the antinociceptive effect of reference analgesics in the Lou/c rat. Br. J. Pharmacol..

[132] Sandvik R.K., Selbaek G., Seifert R., Aarsland D., Ballard C., Corbett A., Husebo B.S. (2014). Impact of a stepwise protocol for treating pain on pain intensity in nursing home patients with dementia: A cluster randomized trial. Eur. J. Pain.

[133] Husebo B.S., Vislapuu M., Cyndecka M.A., Mustafa M., Patrascu M. (2022). Understanding Pain and Agitation Through System Analysis Algorithms in People With Dementia. A Novel Explorative Approach by the DIGI.PAIN Study. Front. Pain Res..

[134] Helvik A.S., Bergh S., Šaltytė Benth J., Borza T., Husebø B., Tevik K. (2023). Pain and quality of life in nursing home residents with dementia after admission—A longitudinal study. BMC Health Serv. Res..

[135] Ai R., Zhuang X.-X., Anisimov A., Lu J.-H., Fang E.F. (2022). A synergized machine learning plus cross-species wet-lab validation approach identifies neuronal mitophagy inducers inhibiting Alzheimer disease. Autophagy.

[136] La Joie R., Visani A.V., Baker S.L., Brown J.A., Bourakova V., Cha J., Chaudhary K., Edwards L., Iaccarino L., Janabi M. (2020). Prospective longitudinal atrophy in Alzheimer’s disease correlates with the intensity and topography of baseline tau-PET. Sci. Transl. Med..

[137] Scuteri D., Rombolà L., Watanabe C., Sakurada S., Corasaniti M.T., Bagetta G., Tonin P., Russo R., Nucci C., Morrone L.A. (2020). Impact of nutraceuticals on glaucoma: A systematic review. Prog. Brain Res..

[138] Bayer A., Tadd W. (2000). Unjustified exclusion of elderly people from studies submitted to research ethics committee for approval: Descriptive study. BMJ.

[139] Scuteri D., Adornetto A., Rombolà L., Naturale M.D., De Francesco A.E., Esposito S., Zito M., Morrone L.A., Bagetta G., Tonin P. (2020). Pattern of triptans use: A retrospective prescription study in Calabria, Italy. Neural Regen. Res..

[140] Scuteri D., Corasaniti M.T., Tonin P., Nicotera P., Bagetta G. (2021). Role of CGRP pathway polymorphisms in migraine: A systematic review and impact on CGRP mAbs migraine therapy. J. Headache Pain.

